# The New York Institute of Stomatology

**Published:** 1905-08

**Authors:** 

**Affiliations:** The New York Institute of Stomatology


### Reports of Society Meetings.

### THE NEW YORK INSTITUTE OF STOMATOLOGY.

A meeting of the Institute was held at the "Chelsea," No. 222 West Twenty-third Street, New York, on Tuesday evening, April 4, 1905, the President, Dr. C. O. Kimball, in the chair.

The minutes of the last meeting were read and approved.

### INCIDENTS OF OFFICE PRACTICE.

*Dr. H. W. Gillett.*—I have here the casts showing the impacted third molar I spoke of at the last meeting. You will note that the first cast shows the tooth lying nearly horizontal with the side of one cusp showing. Packing the angle with guttapercha, as described by Dr. Allis, served to accomplish little more than to force the gum back. A few weeks' work with grass-line wedge resulted in enough upward and backward movement to make it accessible to the forceps.

The next cast illustrates a means of retaining the rubber dam on partially erupted molars. A 6-gauge seamless German silver band from the orthodontia outfit is quickly filled and cemented on the tooth with rapid setting cement, and the turned edge serves to hold the rubber effectually.

I want to show you Dr. Dwight M. Clapp's "rolling" tooth-brush. Note the small size, the short bristles, and the reverse curve of the handle. These points facilitate getting the brush well under the cheek and tongue, the use of the *ends* of the bristles, which is most important, and the wrist motion essential to the rolling motion which Dr. Clapp prescribes.

I find it helpful to demonstrate in the patient's mouth (with a brush to be given them afterwards) the manner in which I wish them to use the brush. With these brushes my patients are obtaining results in the care of their teeth that surpass all my previous experience. I recommend them to your careful consideration as a most valuable adjunct to prophylaxis. Note particularly the smallest size. It is intended for the baby, but is wonderfully effective for cleaning around regulating apparatus, under bridge-work and tipping molars, and the exposed bifurcations of molar roots. For general use I prefer the smaller adult size in the S. grade.

*Dr. D. H. Allis.*—Dr. Gillett refers to the use of gutta-percha after a method of mine. This is somewhat ancient history. It was several years ago that I used it in this manner, and experience has taught me something since that time about the use of gutta-percha. However, I have seen very satisfactory results from this method, but it must be persistently followed up. At any rate, I think we would all rather see these cases of impacted wisdom-teeth come to the other man's practice.

*The President.*—I reported last month the case of a third molar that I am interested in. I have seen the case to-day, and it is progressing favorably. Originally it showed only one cusp above the gum. There was decay in the back of the second molar, which was filled, and also a cavity in the crown of the third molar. This cavity I filled with amalgam, projecting upward in such a way that a wedge could be brought to bear. From time to time I am adding more amalgam and continuing the wedging. I am endeavoring to make the tooth assume a normal position and become useful.

*Dr. F. C. Brush.*—I frequently have occasion to devise and use little appliances that may be useful to others. One of these is a "reinforced wedge." It consists of a piece of cotton-wood, compressed and shaped in the usual way, with a small hole drilled through it. Through this hole is stretched a thick piece of separating rubber, the principle being that after the wood has fully expanded the rubber will be released and will try to resume its original form, thus continuing the pressure. Such a wedge is very effective for use across wide spaces, especially in orthodontia work, where it may be necessary to regain the space of a missing tooth.

*Dr. F. Milton Smith.*—I want to pass around a porte-polisher. Dr. Gaylord sent this one to me, and he has recommended for use with it ordinary wooden shoe-pegs. They can be obtained in various sizes.

I also wish to call attention to two little approximal trimmers that have been very useful to me. They are in Ivory's set.

*Dr. S. H. McNaughton.*—Several years ago it occurred to me, that it might be a good idea to use a combination of Abbey's soft foil and one of the ordinary foils in the same pellet or cylinder, the idea being that Abbey's soft foil is not made cohesive by annealing, while the other foil in the combination is, and that it might have some advantages over an all-cohesive or an all-non-cohesive cylinder. I think it has. In using the combination of the two kinds of foils I use pointed pluggers. In contours where great strength is required it should not be used. It works very much like tin-foil, but it has the cohesive quality. It is particularly useful in starting fillings and in filling against the walls. In compound approximal cavities the occlusal portion should be filled with cohesive gold.

A little point in regard to wedging teeth. A few days ago I had in my chair a little girl who had quite a good-sized cavity in an upper first molar. The adjoining bicuspid was only about one-third erupted, and, suspecting that there might be a cavity in the distal surface of this bicuspid, it was very necessary to wedge, and it seemed as though it would be a troublesome place to wedge. As a sort of starter I excavated the cavity in the molar and filled it about two-thirds full of glutol (a gelatin which has been treated with formaldehyde, and thus made insoluble), and over this packed gutta-percha into the cavity and against the bicuspid. She came back in about a week with the teeth so nicely wedged that I was able to fill the cavity in the bicuspid with gutta-percha without any opening to the occlusal surface.

Dr. LeRoy presented the reprint of a paper, apropos of supernumerary teeth. The paper mentioned some curious instances in different animals, and following this data presented a course of reasoning tending to show that such occurrences were merely anomalous and not examples of reversion.

Dr. F. C. Brush, of New York, read a paper entitled "The Idle Moments of Busy Men."

(For Dr. Brush's paper, see page 528.)

A paper entitled "Cleansing Teeth, as the Subject appears to the Writer," was then presented by Dr. W. M. Whitlock, of New York.

(For Dr. Whitlock's paper, see page 525.)

*Dr. C. F. Allan.*—I should like to have Dr. Eames, who is with us to-night, tell us about some very beautifully made and delicate cleansing instruments he has recently shown me.

*Dr. G. F. Eames.*—I admit that I did show some instruments to Dr. Allan, but had no thought that they were of sufficient importance to provoke discussion. Referring to the porte-polisher mentioned, I would say that I have devised a simple way of taking one made from brass tubing of different sizes, by soldering to one end of the larger size, at desirable angles, a short piece of the smaller tubing. This makes a very good porte-polisher. I have fifteen or twenty of them, and have sticks prepared ready for use of the various desirable shapes, so that I can pick them up without the waste of an instant of time.

I suppose the essayist referred to those patients who come to him for the first time when he mentioned "congenial surroundings." Surely his regular patients would not present in this condition. I do not make it my invariable practice to first cleanse the patient's teeth. It is possible that at times other things may be of more importance.

*Dr. F. Milton Smith.*—It is a constant question with me to know how to get some moments for my own personal use. The days are so full, sometimes far into the night, that a few minutes that actually belong to me are very valuable; any suggestion as to how best to use them is certainly helpful. A suggestion in Dr. Brush's paper that especially recommended itself to me was the having of a memorandum-pad right at one's hand if we don't have some one to jot things down for us. I have found this an exceedingly valuable aid to my memory and a great saving of spare moments.

Regarding the paper of Dr. Whitlock, I did not know that Dr. Whitlock was using a polisher designed by Dr. Smith. His suggestion that cleansing the teeth is the most important operation that we do for our patients, I think I agree with, if the patients would only keep them clean. If they would do this we would be out of business. Regarding the suggestion that he first cleanses the the teeth before anything else, I do not always do this. Never in my practice have I been able to tell beforehand exactly how long it was going to take me to put in a given filling, and for this reason I often remove simply the heavier accumulations at the first sitting. Thereafter the closing minutes of each sitting are used for more careful cleansing and polishing. This obviates the loss of time that is apt to occur by reserving too much time for one filling and not enough for two.

*Dr. Allis.*—Along the line of Dr. Brush's paper, I think a very good way to keep data of all kinds, whether of articles of interest in different journals or other data, is by the use of an index system; then whenever it is desirable to write upon any given subject all your material is at hand.

*Dr. Eames.*—I use a regular card index system for this purpose, the same as is used in the public libraries. I use this not only for literature, but for various other purposes.

*Dr. C. F. Allan.*—I have only one thing to say in regard to the matter at present under discussion, and that is the great advantage it is to every dentist to have a good secretary. I do not mean one who will do what she is told, but one who can look up an article in a magazine, take enough interest in your work to keep track of magazine articles for you, and who is a memory and, in a measure, an intelligence for you. Such a person is invaluable!

*Dr. C. D. Cook.*—I should like to ask a question referring to a suggestion by Dr. Whitlock, that sixty years ago the dentists of that day cleansed their patients' teeth so carefully as to prevent pyorrhœa alveolaris, as we understand it. Now, I do not know that I understand pyorrhœa as others understand it. I do not know that Dr. Riggs understood the difference between pyorrhœa and that recession of the gums due to a deposit of tartar. I believe that disease caused by tartar to be a very different one from what I conceive to be true pyorrhœa, where two or three teeth in different parts of the mouth may be affected with looseness, with no especial deposits of tartar except in minute quantities about the necks of the teeth. I can look back fifty years,—not very keenly, perhaps,—but I do not remember any such disease as I find nowadays, any more than the physician would find diphtheria, for instance, then as now. At any rate, I think there is a very great difference between the two causes of absorption of the alveolar process due to deposits of tartar and true pyorrhœa.

*Dr. H. L. Wheeler.*—It might be interesting, in connection with what Dr. Cook has just said, to state that I have recently been examining quite a number of skulls from prehistoric man, and I am satisfied, from my observations thus far, that this destruction of alveolar process apparently has no relation to the amount of calcic deposit from the saliva, and that it is of a systemic character. I have found skulls where there was a large quantity of calcic deposit, with no apparent injury to the process. I have found a great many cases where the teeth have been extracted and there has been no tilting. I doubt if there is any such thing as an ideal perfect mouth. I find in the cliff-dwellers practically perfect teeth. I have never seen in them evidences of caries nor alveolar absorption, yet never in any skull of these people have I found a case where they were in the ideal relations described by Dr. Bogue and Dr. Angle. The first molars are about the same, but anterior to that point they differ radically, and invariably I have found the centrals, upper and lower, standing edge to edge. In the case of the Peruvians, while there is a great amount of calcic deposit, there is no great destruction of bone. I have a theory that these people did not suffer from pyorrhœa because they were obliged to exercise their teeth in masticating hard food substances. If a tooth-brush can be made to do the same work, you will get the same results.

It is all a question of environment. If this is changed, Nature has to go to work by a slow process to adapt herself to the changed conditions. Things are not created perfect and then degenerate. They are all the time adjusting themselves to their environment.

*The President.*—I should like to say just a word, if I may be permitted to do so, on this subject of pyorrhœa alveolaris. Dr.

Cook has asked a question, and in answer I should like to mention one case that came into my hands some years ago,—that of a boy sixteen years of age with the history of the loss of teeth from pyorrhœa alveolaris. He had lost one of the central incisors and a lower first molar. One of the upper first molars was hanging by a thread. There was not very much tartar around the teeth. The question was what to do. I simply followed out this plan that Dr. Whitlock has spoken of,—a thorough systematic cleansing of the teeth,—and everything has gone well with the boy since. I think Dr. Whitlock, in his paper, alluded to a fact which I stated some years ago. I said then that although I had been practising twenty-five years, I had seen hardly a single case of pyorrhœa alveolaris, and I could not understand why, although judging from the literature on the subject there was apparently so much of it abroad, I had seen so little of it in my practice. Since then more cases have come under my observation, but up to that time I had been working in a practice where it had been the rule to cleanse the teeth first and regularly. The teeth of these patients were cleansed at regular intervals, not because there were great masses of tartar present, but the teeth were searched with these fine examining instruments, under the gums and on every surface, to detect any microscopical particles of tartar; between the upper molars as well as the lower incisors, and back of the last molars; in fact, everywhere. It was a habit of care and patience that my preceptor had insisted upon for himself, and it had borne fruit, so that I cannot help but feel that pyorrhœa alveolaris, as I understand it and have seen it, is a preventable disease entirely by the very simple method of persistent and faithful cleansing of the teeth in the manner in which Dr. Whitlock has described. Our friend Dr. D. D. Smith wants to do it every month, but in my observation there is no hard and fast rule as to the interval of time. This is a point upon which I should take issue with Dr. Smith. I have patients whom I can see once a year and keep their teeth thoroughly in order. I have had one patient whose teeth I did not clean once in fifteen years. I really could not find a trace of tartar in that mouth, and there was no stain even to polish off. It was the only case I have ever seen. I invariably cleanse the teeth first. The intervals of time to be allowed to pass to keep a mouth in perfect condition must be governed entirely by each individual case. For certain ones a year may elapse, while in other cases the patient must be seen every six or four months, or perhaps every month.

*Dr. Brush.*—Regarding this patient who collected so little deposit, I should like to ask if the arches were in perfect condition.

*The President.*—I have not seen this patient for fifteen years, but my impression is that there were lost teeth in both arches.

*Dr. Brush.*—I have noticed that where the arches are unbroken and are normal in shape there is less deposit than where teeth are missing and perhaps others tilted.

*Dr. LeRoy.*—It has been my experience that the greatest amount of calcic deposit occurs after the teeth have been cleansed; also that the teeth that accumulate tartar most rapidly at the neck are those that are bell-shaped. Such teeth are most subject to pyorrhœa.

Regarding the case mentioned by the president, of the boy sixteen years of age with teeth affected by pyorrhœa, was it not possible that this was a case of salivation due to a mercurial treatment?

Regarding the statements that pyorrhœa did not exist in former times, I have been attending meetings for twenty-five years, and it seemed to be as prevalent then as now.

*The President.*—I did not mean to say that pyorrhœa was unknown twenty-five years ago, but that owing to the careful work of my predecessors in my own practice it had been eliminated.

*Dr. Brush.*—In closing the discussion on my paper, there is perhaps but one thing that I might say, and that is, that there was a time when I never used a memorandum of any kind. I prided myself on my memory and ability to carry in my head all the things I needed. Later, an elderly man suggested to me that this was a foolish practice, making a garret of my brain; that the useless lumber should be conveniently stored out of the way, making room for the more important things of the moment.

*Dr. Whitlock.*—In line with the thoughts already expressed, that pyorrhœa is curable by the simple method of thorough cleansing, I wish to mention one or two cases.

A lady was brought to me by her sister, asking if it was necessary for her to lose all her teeth. I examined them. There did not seem to be many cavities, but the teeth were all loose and in some places there was a good deal of tartar. I was convinced from the appearance of her gums that a great deal of the trouble was due to lack of use, and I asked her, as I always ask patients of this kind, if she did not bolt her food. She admitted that she did. I told her that if she would learn to masticate her food properly I could cure her. I cleansed her teeth thoroughly, taking three hours to do it. She carried out her part faithfully. To-day you would never mistrust that there had ever been anything the matter with her mouth, with the exception that before I saw her she had already had one molar extracted, and I think that tooth could have been saved.

A gentleman had a great deal of trouble when he was a young man. He had had his teeth cleansed regularly, and had had a great many fillings inserted. He told me that a few years ago he started the habit of eating only such foods as he could masticate. He did not eat soup nor ice-cream; in fact, nothing that did not require the use of his teeth. For two years he has not had a filling to be inserted, and, although a smoker, his teeth have not been stained with tobacco.

I have among my patients a young man who bolts his food. Though he brushes his teeth three times daily, it is simply impossible for me or any one else to keep his teeth even in a fairly decent condition. I always impress my patients with the necessity of using their teeth for masticating.

Adjourned.

FRED. L. BOGUE, M.D., D.D.S.,
*Editor The New York Institute of Stomatology.*

